# Critical Role of Endoplasmic Reticulum Stress in Cognitive Impairment Induced by Microcystin-LR

**DOI:** 10.3390/ijms161226083

**Published:** 2015-11-25

**Authors:** Fei Cai, Jue Liu, Cairong Li, Jianghua Wang

**Affiliations:** 1 Department of Pharmacology, Hubei University of Science and Technology, Xianning 437100, China; cf@mail.hbust.com.cn; 2 Department of Pharmacy, The Central Hospital of Wuhan, Tongji Medical College, Huazhong University of Science and Technology, Wuhan 430014, China; whzxyy1880@zxhospital.com; 3 Hubei Province Key Laboratory on Cardiovascular, Cerebrovascular, and Metabolic Disorders, Hubei University of Science and Technology, Xianning 437100, China; lcr@mail.hbust.com.cn; 4 Fisheries College, Huazhong Agricultural University, Wuhan 430070, China

**Keywords:** microcystin-LR, endoplasmic reticulum stress, cognitive impairment, tauroursodeoxycholic acid

## Abstract

Recent studies showed that cyanobacteria-derived microcystin-leucine-arginine (MCLR) can cause hippocampal pathological damage and trigger cognitive impairment; but the underlying mechanisms have not been well understood. The objective of the present study was to investigate the mechanism of MCLR-induced cognitive deficit; with a focus on endoplasmic reticulum (ER) stress. The Morris water maze test and electrophysiological study demonstrated that MCLR caused spatial memory injury in male Wistar rats; which could be inhibited by ER stress blocker; tauroursodeoxycholic acid (TUDCA). Meanwhile; real-time polymerase chain reaction (real-time PCR) and immunohistochemistry demonstrated that the expression level of the 78-kDa glucose-regulated protein (GRP78); C/EBP homologous protein (CHOP) and caspase 12 were significantly up-regulated. These effects were rescued by co-administration of TUDCA. In agreement with this; we also observed that treatment of rats with TUDCA blocked the alterations in ER ultrastructure and apoptotic cell death in CA1 neurons from rats exposed to MCLR. Taken together; the present results suggested that ER stress plays an important role in potential memory impairments in rats treated with MCLR; and amelioration of ER stress may serve as a novel strategy to alleviate damaged cognitive function triggered by MCLR.

## 1. Introduction

There has been increasing attention on toxic blooms of cyanobacteria over the past few decades. Microcystins (MCs) are the most commonly reported cyanotoxins all over the world, with molecular weights ranging between 900 and 1100 Da [1,2,3]. Cyanobacteria-derived microcystin-leucine-arginine (MCLR) is considered to be the most abundant and most toxic analogue of MCs [4].

Previous reports from field and laboratory work have indicated that liver is the main accumulation organ after MCs exposure [5,6]. Abundant proof from aquatic and terrestrial animals confirmed that MCs can cross the blood-brain-barrier to induce neurotoxicity [7,8,9,10]. Neurological symptoms, such as dizziness, tinnitus, vertigo visual disturbance, have been reported in patients intravenously exposed to MCLR [11,12]. In mammals, memory impairment has been observed in rats after acute or sub-chronic exposure to MCLR [13,14]. Moreover, progestational exposure to microcystin-LR caused cognitive impairment in rat offspring [15]. Long-term potentiation (LTP) is a well-known model to study the cellular mechanism of synaptic plasticity, which related to memory function [16]. Although deficiencies in LTP have been reported in our previous study [17], which could help explain cognitive deficit in rats exposed to MCLR, the detailed mechanisms underlying the chain reaction are still elusive.

It is generally believed that primary mechanism of MCLR toxicity is through the specific inhibition of protein phosphatase 1 (PP1) and 2A (PP2A) [18]. A lot of other factors have been suggested to be involved, such as caspase-dependent apoptosis, increased oxidative stress, microtubule-associated tau protein hyperphosphorylation and destruction of cytoskeletal structures [19,20,21]. Endoplasmic reticulum (ER) stress, is a defense system that deals with the accumulation of unfolded proteins in the ER lumen. Though modest ER stress could prevent the damage caused by stress, long-term or serious stress response induces apoptosis and may play an important role in the pathogenesis of many diseases [22]. The activation of c-Jun NH_2_-terminal kinase (JNK), transcriptional induction of CCAAT-enhancer-binding proteins (C/EBP) homologous protein (CHOP) and/or caspase 12 dependent pathways could promote the ER-dependent apoptotic processes [23]. The hippocampus is an important brain region susceptible to stress and plays a vital role in learning and memory. In our previous study, MCLR was found to induce spatial memory deficit, histological and ultrastructural injuries, serious oxidative damage, as well as neuronal apoptosis in hippocampus of rats [14]. Recent work from our laboratory identified that MCLR could up-regulate the expression of 78-kDa glucose-regulated protein (GRP78) [13]. As increased expression of GRP78 is a good marker for ER stress [24], we hypothesized MCLR may cause severe ER stress that is related to neuronal apoptosis in the hippocampus, thereby causing cognitive impairment.

In this study, we studied for evidence about MCLR-induced ER stress in the hippocampus and its role in underlying synaptic plasticity and cognitive impairment. Lots of reports have indicated that tauroursodeoxycholic acid (TUDCA) is an effective blocker of ER stress [25,26]. In our research, dysfunction of cognition and synaptic plasticity could be prevented by TUDCA, suggesting that ER stress plays a critical role in underlying memory deficits in rats treated with MCLR.

## 2. Results

### 2.1. ER Stress Inhibitor Rescued MCLR-Induced Memory Impairment

**Figure 1:**
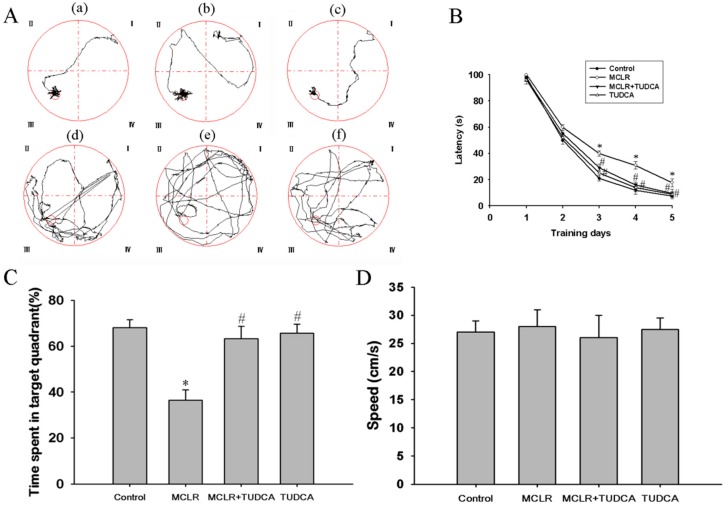
TUDCA rescued MCLR-induced damage of memory and learning in rats. All rats were submitted to five days training to remember the location of a hidden platform in behavioral test. (**A**) The typical swimming-tracking path of rats; **a**–**c** are the path on the last training day; **d**–**f** are the path in the probe trial test; **a** and **d**: control group; **b** and **e**: MCLR-treated group; **e** and **f**: TUDCA-treated group; (**B**) Mean latencies to find the hidden platform; (**C**) Time spent in target quadrant in the probe trial on the sixth day; (**D**) The average swimming speed of rats during the acquisition and probe trial. Values represent mean ± SEM. Each group has 10 animals. * *p* < 0.05 compared with control group, # *p* < 0.05 compared with MCLR alone group.

**Figure 2:**
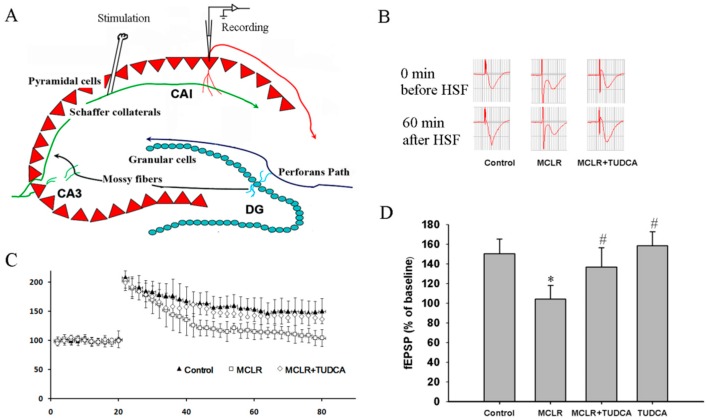
MCLR restored hippocampal LTP inhibition in MCLR-treated rats. (**A**) An ideographic electrophysiology recording with stimulating electrode and recording electrode placed in the Schaffer collaterals and the stratum radiatum layer of the CA1 region, respectively; (**B**) Representative traces of fEPSP before and after HFS; (**C**) MCLR impaired the magnitude of LTP, reflected as a decrease in the average potentiation of fEPSP in 60 min after LTP induction; (**D**) Histograms indicated administration of TUDCA during MCLR treatment rescued the impaired LTP induced by MCLR. * *p* < 0.05 compared to control, # *p* < 0.05 compared to MCLR alone, One-way ANOVA (*n* = 15 for control group, *n* = 13 for MCLR group, *n* = 13 for MCLR + TUDCA group and *n* = 14 for TUDCA group) followed by the Duncan multiple group comparison. Values represent mean ± SEM.

Firstly, we tested the effects of MCLR on spatial learning and memory function using a Morris water maze. MCLR treatment has no effect on water and body weight, food intake, and overall mobility of rats (data were not shown). As shown in Figure 1A, we found that MCLR-treated rats took longer to find the visible platform than the control group from typical swimming paths in the acquisition trials. Figure 2B showed the mean escape latency with training. Daily training had a significant main effect on both factors disclosed by a Two-Way Analysis of Variance (ANOVA): group (F(3,27) = 8.14, *p* < 0.05) and day (F(4,104)) = 8.26, *p* < 0.05). MCLR-treated rats had significantly more escape latency than the control group. However, the poor performance was alleviated by treatment with ER stress inhibitor, TUDCA (*p* < 0.05, *post hoc* Duncan’s test). The swimming paths (Figure 1A) and the time spent in target quadrant in the probe trials were used to evaluate performance. Compared with control rats, MCLR-treated rats spent significantly less time in the target quadrant, while co-administration of TUDCA significantly ameliorated the performance (*p* < 0.05 compared with MCLR alone, *post hoc* Duncan’s test) and had no difference from the control group (*p* > 0.05, Figure 1C).No significant differences of swimming speed were observed among three groups (Figure 1D).

### 2.2. MCLR-Caused ER Stress Impaired Hippocampal Synaptic Plasticity

We next tested if MCLR could affect long-term potentiation (LTP) in the Schaffer collateral-CA1 pathway in the hippocampus. As shown in Figure 2B,C, we found that preincubation with 0.4 μM MCLR (30 min) decreased the magnitude of tetanus-induced (100 Hz, 1 s) LTP in this pathway. The magnitude of fEPSP was 104.3% ± 14.1% of the baseline (13 slices from five rats), which was significantly smaller compared with control (150.4% ± 15.2%, 15 slices from five rats, *p* < 0.05). However, pretreatment with TUDCA significantly mitigated the inhibition of the LTP (137.3% ± 18.2%, 13 slices from four rats, *p* < 0.05 compared with MCLR alone). The effect of TUDCA is specific to MCLR-induced impairment in LTP because TUDCA itself did not change the magnitude of LTP (Figure 2D, 14 slices from four rats, *p* > 0.05 compared with control).

### 2.3. MCLR induced ER Stress in Hippocampus of Rats

**Figure 3:**
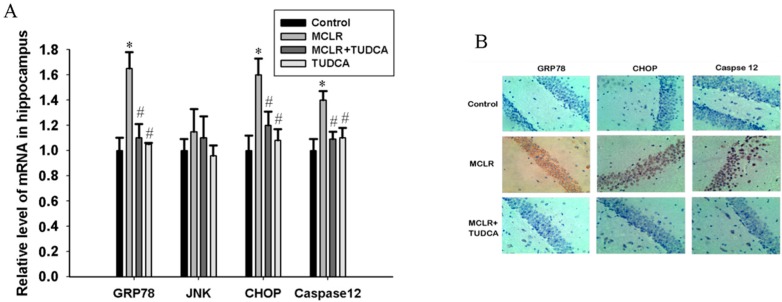
MCLR exposure caused ER stress in the hippocampus of rats. (**A**) Relative mRNA levels of GRP78, JNK, CHOP, and caspase 12. Data are expressed as the mean ± SEM of results from four individual experiments. * *p* < 0.05 compared to control, # *p* < 0.05 compared to MCLR alone; (**B**) Histological sections of brains in control, MCLR and MCLR&TUDCA group were stained with GRP78, CHOP, and caspase 12 antibodies under a light microscope (200×).

To confirm the role of ER stress in the brain under MCLR exposure, we detected the mRNA and protein expression of the ER stress makers using RT-PCR analysis and immunohistochemistry method. As shown in Figure 3A, MCLR exposure significantly increased the mRNA expression levels of GRP78, CHOP and caspase 12 compared to control, but JNK mRNA expression had no significance difference between control and MCLR exposure rats. Meanwhile the up-regulation of GRP78, CHOP and caspase 12 mRNA expression were effectively inhibited by TUDCA treatment (*p* < 0.05, *post hoc* Duncan’s test). However, TUDCA itself did not alter the mRNA expression level of these three molecular (*p* > 0.05 compared with control).

Consistent with the up-regulation of GRP78, CHOP, and caspase 12 mRNA expression, we demonstrated that the expression of GRP78, CHOP, and caspase 12 protein levels were also increased in the hippocampus of rats exposed to MCLR (Figure 3B). However, the significantly higher expression of these three proteins were significantly decreased when co-treatment with TUDCA.

### 2.4. MCLR Induced Ultrastructural Changes and Apoptosis in Hippocampus

**Figure 4:**
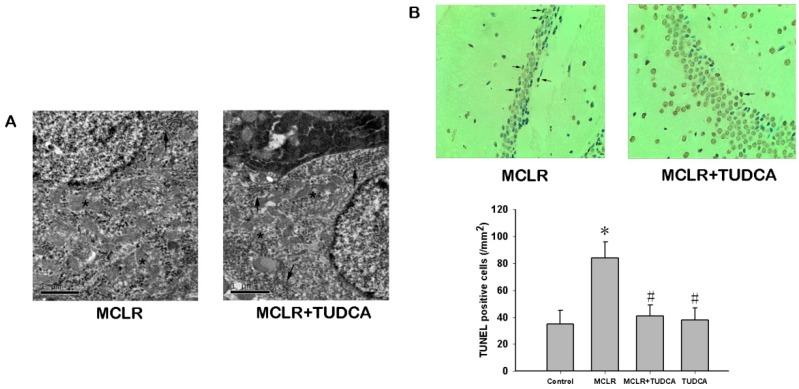
MCLR induced ultrastructural changes and apoptosis in the hippocampus. (**A**) Transmission electron micrographs of hippocampus in the MCLR and MCLR&TUDCA group. Most of the perinuclear ER (indicated by arrows) in MCLR group were misoriented, swollen, and distorted. No obvious changed were observed in the mitochondria (indicated by stars). Treatment with TUDCA rescued the alteration in ER structure; Scale bar, 1 µm; (**B**) TUNEL staining showed TUDCA could rescue the apoptotic neuronal death triggered by MCLR. Apoptotic cell were pointed by arrows (200×). Data are expressed as the mean ± SEM of results from four individual experiments. * *p* < 0.05 compared to control, # *p* < 0.05 compared to MCLR alone.

We further analyzed the ultrastructural of ER to obtain more evidence of ER stress in the hippocampus of rats treated with MCLR. Significant alterations in ER ultrastructure in CA1 neurons form rats exposed to 14 days of MCLR were observed by electron microscopy. As shown in Figure 4A, swollen and distorted ER was found in MCLR-treated group, and the ER lost the typical parallel arrangement. The pathological appearance of ER could be prevented by treatment with TUDCA in the hippocampal CA1 neurons.

TUNEL staining has become one of the main methods for detecting apoptotic programmed cell death, and exhibits the biochemical hallmark of apoptosis, internucleosomal DNA fragmentation. Apoptotic cells within hippocampal CA1 region exhibiting DNA damage were stained brown (Figure 4B). The number of positive cells was significantly higher in the MCLR-treated rats compared to the control rats in hippocampal CA1 and DG region (Figure 4, *p* < 0.05), which were alleviated by TUDCA treatment (*p* < 0.05).

## 3. Discussion

In this study, a systematic and comprehensive investigation was performed to pinpoint the critical role of ER stress in potential memory damage induced by MCLR exposure. First, at the behavioral level, co-administration of TUDCA and MCLR recused acquisition of spatial memory that was impaired by prolonged MCLR. Second, at the neuronal circuit level, inhibition of ER stress by TUDCA alleviated the injury in hippocampal long-term potentiation of rats caused by MCLR. Third, we found that 14 days of exposure to MCLR lead to the pathological appearance of ER, which could be prevented by treatment with TUDCA. Meanwhile MCLR could up-regulate the expression of GRP78, caspase 12, and CHOP, three key hallmarks of ER stress. Finally, MCLR induced apoptosis in the hippocampal CA1 subfield and the effects were mitigated by inhibition of ER stress. Overall, these data demonstrate that a primary event in MCLR-triggered memory dysfunction is the induction of the ER pathway in the hippocampus, which leads to alteration of synaptic plasticity as well as apoptosis.

Several previous studies have reported rats exposed to MCLR showed deficits in hippocampal-dependent learning and memory function [13,14,15,17]. Our previous results demonstrated that MCLR induced cognitive impairment as assessed by the Morris water maze, and inhibition of ER stress blunted cognitive deficit. LTP, known as a notable form of hippocampal synaptic plasticity, has been established as the prominent synaptic model for studying the molecular and cellular mechanism of learning and memory [16]. To further examine the possibility of ER stress might be involved in the cognitive deficit of rat exposed to MCLR, we explored the effect of TUDCA, an endogenous bile acids derivative, on the regulation of LTP by MCLR. TUDCA was previously shown to regulate ER function defending from UPR activation and ER stress-related apoptosis by exhibiting directly on its signaling pathway [25,26]. The present study showed TUDCA could rescue LTP impairment induced by MCLR. Our results further supported the hypothesis that ER stress is responsible for the cognitive impairment caused by MCLR although we could not identify if this mediation might be a direct result or a second event. Further investigation is needed for a more precise description of the mechanism.

Accumulating evidence demonstrated that ER stress is involved in toxicity of MCLR. ER stress is promoted by disturbance in ER homeostasis, leading to the activation of GRP78, which dissociated from the three major ER sensors to launch UPR [27]. Prolonged and/or serious ER stress causes apoptosis, which is regulated by CHOP and/or a caspase 12-dependent signaling pathway [22]. In multiple cell types, especially hepatic cells, MCLR exposure causes distention of the ER accompanied by mitochondrial damage and nucleolus deformation [28,29]. Moreover, MCLR significantly decreased the GRP78 level and increased the expression of CHOP and activation of caspase 12 [28,29,30,31]. The proteomic results revealed that perinatal exposure to MCLR could remarkably up-regulate the abundance of GRP 78 in the brain of neonatal rats [32], consistent with our previous study on the rats that received ip injection of MCLR for 50 days [13]. To investigate ER stress following MCLR treatment to rats, we examined the expression of specific hallmarks of ER stress, GRP78 and CHOP. Our research showed the mRNA and protein expression levels of GRP78 and CHOP were increased in rats exposed to MCLR. CHOP could decrease expression of the anti-apoptotic molecules and increase expression the expression of pro-apoptotic molecules to trigger apoptotic cell death [33]. ER-related apoptotic cell death distinguishes from the intrinsic pathway because induction of caspase 9 can be activated directly by caspase 12, without the releasing of cytosolic cytochrome c from mitochondria [25]. In our study, up-regulation of GRP78, CHOP, and caspase 12, paralleled with the increased apoptotic cell in the hippocampus of rats exposed to MCLR, which supported the idea that MCLR induces apoptotic neuronal death in hippocampus through induction of ER stress pathway.

## 4. Experimental Section

### 4.1. Animals

Three-month-old male Wistar rats with the weights 180–200 g were obtained from Hubei Laboratory Animal Center (Certificate No. SCXK[E]2008-0005, Wuhan, China). These rats were housed in an environment of constant temperature at (25 ± 2 °C) and relative humidity (60% ± 10%). They received standard rodent diet and tap water ad libitum and maintained on a 12-h light/dark cycle. All experimental procedures were approved by Animal Experimentation and Ethics Committee of Huazhong Agricultural University. The rats were randomly divided into three groups. The MCLR-treated group received intraperitoneal injection of MCLR (10 μg/kg/day for 14 days), the TUDCA-treated MCLR group received intraperitoneal injection of MCLR and TUDCA (100 mg/kg/day for 14 days), and the control group injected an equivalent volume of saline solution.

### 4.2. Chemicals

Purified MCLR (purity ≥ 98%) was purchased from Alexis Biochemicals (Lausen, Switzerland). TUDCA was obtained from Sigma-Aldrich (St. Louis, MO, USA). MCLR and TUDCA were dissolved in saline and diluted to obtain final concentration. Anti-GRP78, anti-CHOP, and anti-caspase 12 were purchased from Cell Signaling Biotechnology (Danvers, MA, USA). All other agents were obtained from commercial sources.

### 4.3. Behavioral Studies

The spatial learning and memory function of rats was investigated by the Morris Water Maze experiment [34]. Animals swam in a metal circular pool (120 cm in diameter, 80 cm tall), in which a circular Plexiglas platform (10 cm in diameter, 40 cm tall) was hidden 1–2 cm below the surface of the water (26 ± 1 °C). Training and testing procedure were conducted as previous described [13,14].The training session consisted of four trials in total (one trial per quadrant) with a 30 s inter-trial interval. Trials were performed once per day for five days. During training, rats were placed in one of four randomly chosen locations facing the wall of the tank and were allowed to search for the hidden platform for 60 s. If the rat failed to find the platform, it was placed on the platform by the experimenter. As the rats swam around the pool, various parameters, such as the time taken to reach the platform and swimming paths, were record by a video imaging analysis system. On the sixth day, a probe testing trial was performed to measure the retention of spatial memory when the platform was removed. The time spent in the target quadrant was recorded.

### 4.4. LTP Measurements

The electrophysiological recording procedure was performed as described in our previous study [35]. Briefly, brains were immediately removed and immersed in ice-cold artificial cerebrospinal fluid (ACSF) containing (in mM) 119 NaCl, 3.5 KCl, 1.3 MgSO_4_, 2.5 CaCl_2_, 1 NaH_2_PO_4_, 26.2 NaHCO_3_ and 11 glucose. Four hundred-micrometer-thick coronal brain slices containing hippocampus were cut using a vibrating-blade microtome. Field excitatory postsynaptic potentials (fEPSP) were evoked by constant stimulation in the Schaffer collaterals with a bipolar electrode and recorded in the stratum radiatum layer of CA1 with a glass micropipette filled with 3 M NaCl. High-frequency stimulation (HFS: 100 Hz for 1 s; 30 s interval) was applied to induce LTP, which measured by the magnitude of fEPSP.

### 4.5. Real-Time Polymerase Chain Reaction

Total RNA was isolated from hippocampus using TRIzol reagent according to the manufacturer’s protocol (Invitrogen, Carlsbad, CA, USA). The synthesis of first-strand cDNA, and real-time PCR were performed following the method described in our previous study [13]. The PCR cycle number that crosses the signal threshold (*C*_t_) was used to define the endpoint in the real-time PCR quantification. The comparative *C*_t_ method was performed to detect the quantification of target gene expression. All of the values were normalized to GAPDH expression levels. According to the previous study [36], the primers of tested genes were listed in Table 1.

### 4.6. Immunohistochemistry Assay

Brains were embedded in paraffin, and transverse paraffin sections containing hippocampus (4 mm thickness) were mounted in saline-coated slides. For immunohistochemistry, the sections were washed in 0.01 M PBS containing 0.3% Triton X-100 followed by immersing in 10% goat serum in PBS for 2 h at 37 °C, then incubated with primary antibody at 4 °C overnight in PBS containing 1.5% normal goat serum. Sections were subsequently incubated with biotinylated goat anti-rabbit IgG secondary antibody for 2 h at room temperature. Then, the secondary antibody was rinsed, and the sections were labeled with avidin-peroxidase complex reagent (ABC kit; vector) for 1 h. The sections were developed with 3,3′-diaminobenzidine (DAB). As a control, the primary antibodies were replaced with non-immune IgG and processed in parallel.

**Table 1:** Primer sequences used for real-time PCR in this study.

Gene Name	Primer Sequence (5′–3′)	Accession No.	Melting Temperature (°C)	Product Size (bp)
*GRP78*	F-AACCCAGATGAGGCTGTAGCA	NM022310	60	91
	R-ACATCAAGCAGAACCAGGTCAC			
*JNK*	F-TGATGACGCCTTACGTGGTA	XM341399	60	114
	R-GGCAAACCATTTCTCCCATA			
*CHOP*	F-CCAGCAGAGGTCACAAGCAC	NM007837	60	126
	R-CGCACTGACCACTCTGTTTC			
*Caspase12*	F-CACTGCTGATACAGATGAGG	NM130442	60	138
	R-CCACTCTTGCCTACCTTCC			

### 4.7. TUNEL Assay

Terminal deoxynucleotidyl transferase-mediated (dUTP) nick end-labeling (TUNEL) staining of paraffin-embedded section of hippocampi was carried out using Roche *in situ* death detection kit (Roche Diagnostics GmbH, Mannheim, Germany) following the manufacturer’s instructions. Four-micrometer sections were then incubated with proteinase K (20 μg/mL in 10 mM Tris-Cl, pH 7.6, for 15 min at room temperature), blocked in 3% H_2_O_2_ in methanol for 10 min, permeabilized for 2 min in 0.1% Triton X-100/sodium citrate at 4 °C, and treated with TUNEL reaction mixture. Improvision Open Lab version 3.1.6 software was used to observe and analyze the slides.

### 4.8. Ultrastructural Observation

The hippocampus was dissected and rinsed in PBS, post fixed flat in 1% osmium tetroxide for 30 min and dehydrated in ascending concentrations of ethanol. Tissues were then treated with propylene oxide and impregnated with resin overnight (Durcupan ACM; Sigma, St. Louis, MO, USA). Each hippocampus was embedded in Epon 812 for 1 h at 60 °C and polymerized overnight at 80 °C. The tissues were sliced into a series of consecutive ultrathin sections (60–70 nm) using a Leica Ultra-Cut knife (Leica, Wetzlar, Germany), stained with uranyl acetate-lead citrate. The section was examined under a FEI Tecnai G212 (FEI company, Eindhoven, The Netherlands) transmission electron microscope at 10,000–20,000× magnification.

### 4.9. Statistical Analysis

The results were expressed as means with standard errors of the mean (SEM). Statistical analyses were performed using SPSS 16.0. Group differences in the escape latency in the Morris water maze training task were analyzed using two-way ANOVA (*n* = 10 for each group). One-way ANOVA followed by the Duncan multiple group comparison was used to analyze group differences of the data, *p* < 0.05 was considered statistically significant.

## 5. Conclusions

In conclusion, the present study demonstrated that induction of ER stress and associated increased apoptosis in hippocampus play a critical role in cognitive impairment induced by MCLR exposure. Inhibition of ER stress by chemical blockers such as TUDCA is effective in alleviating the damage at the cellular and behavioral levels.
